# Differences in learning characteristics between support vector machine and random forest models for compound classification revealed by Shapley value analysis

**DOI:** 10.1038/s41598-023-33215-x

**Published:** 2023-04-12

**Authors:** Friederike Maite Siemers, Jürgen Bajorath

**Affiliations:** B-IT, LIMES Program Unit Chemical Biology and Medicinal Chemistry, Department of Life Science Informatics and Data Science, Rheinische Friedrich-Wilhelms-Universität, Friedrich-Hirzebruch-Allee 5/6, 53115 Bonn, Germany

**Keywords:** Cheminformatics, Computational chemistry

## Abstract

The random forest (RF) and support vector machine (SVM) methods are mainstays in molecular machine learning (ML) and compound property prediction. We have explored in detail how binary classification models derived using these algorithms arrive at their predictions. To these ends, approaches from explainable artificial intelligence (XAI) are applicable such as the Shapley value concept originating from game theory that we adapted and further extended for our analysis. In large-scale activity-based compound classification using models derived from training sets of increasing size, RF and SVM with the Tanimoto kernel produced very similar predictions that could hardly be distinguished. However, Shapley value analysis revealed that their learning characteristics systematically differed and that chemically intuitive explanations of accurate RF and SVM predictions had different origins.

## Introduction

In pharmaceutical research, machine learning (ML) methods have become increasingly important to address challenging tasks including, among others, the identification of drug-like active compounds, computer-aided synthesis and reaction prediction, or de novo compound design^1–6^**.** Despite progress made with many applications in these and other areas, the acceptance of ML predictions in interdisciplinary research continues to be limited due to the black box character of most ML approaches^7^. The lack of transparency of model decisions often restricts the trust in predictions and, consequently, the impact on experimental design^7,8^. To address this problem, concepts from explainable artificial intelligence (XAI) can be considered that make it possible to better understand ML models and rationalize their predictions^9–12^.

While different explanation methods can be considered for ML in chemistry and drug discovery, model interpretation is still far from being routine and more of an exception than the rule. Relevant XAI approaches can be confined to a particular ML algorithm or generally applicable (model-agnostic). Typically, such approaches aim to reveal the global relevance of representation features for an ML model or provide explanations of individual predictions. For example, feature attribution methods such as Local Interpretable Model-Agnostic Explanations (LIME)^13^, Deep Learning Important Features (DeepLIFT)^14^ or Shapley values^15^ and their local ML approximation termed Shapley Additive Explanations (SHAP)^16^ estimate the relevance of each input feature for a given prediction. Instance-based model interpretation aims to identify features whose presence is essential for a given prediction or whose absence inverts a prediction as assessed, for example, through counterfactuals^17^ or contrastive explanations^18^. Furthermore, graph-based approaches attempt to explain message passing mechanisms and identify edges and/or nodes that are decisive for predictions^19–21^. In self-explaining neural networks, model architecture is altered to ensure feature interpretability^22^. Moreover, uncertainty estimation quantifies confidence levels or expected errors in predictions^23,24^ and is closely related to XAI. Model-agnostic methods such as LIME or SHAP (which are conceptually related) make it also possible to compare predictions using different ML methods. However, such comparisons are currently still rare.

In this work, we have carried out large-scale compound activity predictions using random forest (RF)^25^ and support vector machine (SVM)^26^ models derived on the basis of training sets of increasing size and analyzed the results in detail to better understand their learning characteristics. The study was specifically designed to compare and explain sets of binary classification models based upon these algorithms for distinguishing between different combinations of compound activity classes. To these ends, we have extended the Shapley value formalism through the calculation of cumulative instance- and feature-based variants of exact Shapley values, feature contribution patterns, and scores derived from Shapley values.

## Results

### Scope of the analysis

RF and SVM models are widely used for compound classification and activity prediction. We have carried out systematic activity-based compound classification for all 21 pairwise combinations of seven compound activity classes that each contained more than 1000 qualifying compounds after applying high confidence data selection criteria (see “Methods”), as summarized in Table 1. This threshold was chosen since XAI analysis of classification models required large numbers of source compounds. In addition to containing more than 1000 compounds, the selected activity classes were required to yield comparable intra- and inter-class similarity values to exclude chemically distinct classes from compound classification studies (see “Methods”). Targets of the selected activity classes included different enzymes and receptors (Table 1).

**Table 1:** Compound activity classes.

Number	Target name	ChEMBL ID	Compounds
1	Acetylcholinesterase	220	1229
2	Beta-secretase 1	4822	1192
3	Cyclooxygenase-2	230	1090
4	Epidermal growth factor receptor erbB1	203	1281
5	Hepatocyte growth factor receptor	3717	1299
6	MAP kinase p38 alpha	260	1309
7	Vascular endothelial growth factor receptor 2	279	1988

RF and SVM classifiers were derived to distinguish between compounds from different activity classes on the basis of the same training sets of increasing size, thus enabling direct comparison of the predictions and providing a meaningful basis for model explanation. For the comparison of predictions based on training sets of increasing size, constantly sized test sets were required for all combinations of activity classes, with equal contributions from each class (see “Methods”). For both methods, the importance of features that were present or absent in test compounds for their predictions was quantified using exact Shapley values^15^ and prediction and feature contribution patterns were determined and compared. Notably, different from other feature weighting methods, Shapley values have the principal advantage that they not only quantify the contributions of features present but also absent in test instances^15,16^, which was found to be of critical relevance for our analysis, as described below.

### Prediction accuracy

**Figure 1:**
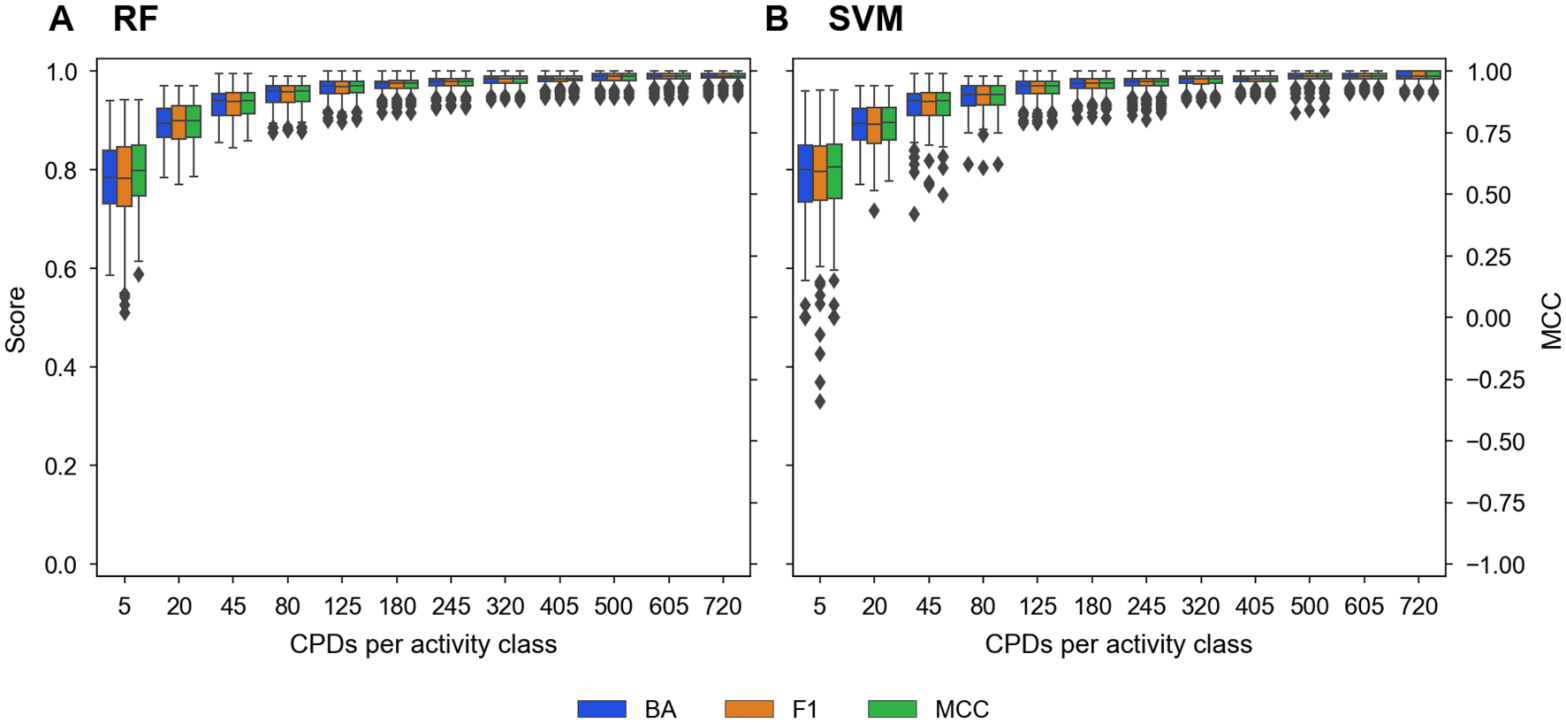
Prediction accuracy**.** For (**A**) RF and (**B**) SVM models built on the basis of training sets of increasing size (CPDs per activity class; x-axis), the distribution of prediction accuracy values is reported in boxplots using BA and F1 scores (y-axis on the left) and MCC values (y-axis on the right). In boxplots, the median value is represented by the horizontal line, and the box defines upper and lower quantile. Upper and lower whiskers represent the maximum and minimum value, respectively. Diamond symbols mark statistical outliers.

For each activity class, 12 differently-sized training sets were generated. For a given pair of activity classes, the smallest training set contained 10 compounds (five from each class) and the largest set 1440 compounds (720 from each class). The sizes of all 12 training sets are reported in Fig. 1. For each training set size, ten different sets were randomly selected for ten independent prediction trials. Hence, a total of 5040 models were derived (2520 models each for RF and SVM) and evaluated using identical test sets comprising 200 compounds (100 from each class) on the basis of the Matthew’s correlation coefficient (MCC), balanced accuracy (BA), and F1-score (F1) performance metrics (see “Methods”).

Figure 1 reports the distribution of prediction accuracy values for RF (Fig. 1A) and SVM models (Fig. 1B) and training sets of increasing size over all activity class pairs. In accord with earlier observations^27^, the performance of the models generally increased with increasing training data volumes. However, models derived from the smallest training data sets were already predictive, with median BA/F1 and MCC values of ~ 0.8 and ~ 0.6, respectively. Small training sets yielded broad value distributions, reflecting variable prediction outcomes. With increasing training set size, the predictions became stable, as indicated by narrow distributions, essentially reaching a plateau with nearly optimal performance for both RF and SVM models based upon training sets comprising 250 compounds, as indicated by median MCC, BA, and F1 values of $0.97$, $0.98$, and $0.98$, respectively. For a comparative analysis of model decisions, these predictions provided an excellent basis.

### Prediction patterns

For comparing RF and SVM predictions, we introduced *prediction* *patterns* that were assessed for each test compound in each trial. For comparison, prediction patterns were consistently defined for RF and SVM models. A prediction pattern consisted of a vector with one entry (bit) for each training set size. A bit setting of 1 and 0 indicated a correct and incorrect prediction, respectively. For a given prediction trial, the corresponding test set was used to assess all models (see “Methods”). Prediction patterns were classified as follows:

1. *Consistently* *correct*: The test compound was correctly predicted by all models.
2. *Consistently* *incorrect*: The compound was incorrectly predicted by all models.
3. *Start:* *correct,* *End:* *incorrect*: The compound was correctly predicted for small training set sizes and incorrectly predicted when training set sizes increased.
4. *Start:* *incorrect,* *End:* *correct*: The compound was incorrectly predicted for small training set sizes and correctly predicted when training set sizes increased.
5. *Variable*: The compound was inconsistently predicted across different models.

Table 2 reports the counts of different prediction patterns for RF and SVM models and their exact intersection. *Consistently* *correct* prediction patterns represented by far the largest amount, followed by *Start:* *incorrect,* *End:* *correct* patterns. Only few compounds (consistently less than 100 of 5014 unique test compounds) yielded unexpected *Start:* *correct,* *End:* *incorrect* or *Consistently* *incorrect* patterns. *Variable* patterns were more frequently observed, for more than 1000 compounds for both RF and SVM. The generally large intersections between patterns reflected similar prediction phenotypes for RF and SVM, as has also been apparent in Fig. 1. Hence, RF and SVM predictions were globally very similar and nearly indistinguishable.

**Table 2:** Prediction patterns. Counts of prediction patterns and corresponding compounds are reported for RF and SVM.

Prediction pattern name		RF	SVM	Intersection	%
Consistently correct	#Patterns	$\mathrm{30,865}$	$\mathrm{30,003}$	$\mathrm{27,318}$	$91.05$
	#CPDs	$4806$	$4794$	$4654$	$97.08$
Consistently incorrect	#Patterns	$187$	$136$	$107$	$78.68$
	#CPDs	$83$	$70$	$54$	$77.14$
Start: correct End: incorrect	#Patterns	$50$	$71$	$13$	$26.00$
	#CPDs	$40$	$55$	$11$	$27.50$
Start: incorrect End: correct	#Patterns	$7894$	$7977$	$3861$	$48.91$
	#CPDs	$2735$	$2958$	$1878$	$68.67$
Variable	#Patterns	$3004$	$3813$	$467$	$15.55$
	#CPDs	$1480$	$1785$	$386$	$26.08$

The intersection column gives the number of exactly matching patterns for RF and SVM (followed by the percentage).

However, prediction patterns also revealed differences in learning characteristics between RF and SVM, for example, the small intersection of the *Variable* and *Start: correct, End: incorrect* patterns. Furthermore, for the more frequently observed *Start: incorrect, End: correct* pattern, the intersection was also only ~ 50%.

### Rationalizing predictions

Given that RF and SVM models produced very similar predictions for the 21 pairs of activity classes, we investigated whether or not corresponding features were responsible for these predictions and whether RF and SVM had similar learning characteristics, despite algorithmic differences. Therefore, we carried out a comparative Shapley value analysis (see “Methods”) across all models. For decision tree methods such as RF and SVM employing the Tanimoto kernel, exact Shapley values can be calculated using the TreeExplainer^28^ and Shapley Value-Expressed Tanimoto Similarity (SVETA)^29^ methods, respectively (instead of locally approximated values as for other ML methods using SHAP^16^). For SVM, the use of the Tanimoto kernel was mandatory to enable the calculation of exact Shapley values (which is currently not possible for other kernels)^29^. Approximated SVM Shapley values only poorly correlated with exact values^29^, which was insufficient for accurate model explanation. For comparison, SVM compound classification was repeated with an alternative (RBF) kernel, yielding nearly indistinguishable prediction accuracy compared to the Tanimoto kernel (Supplementary Fig. S1).

### Features determining predictions

**Figure 2:**
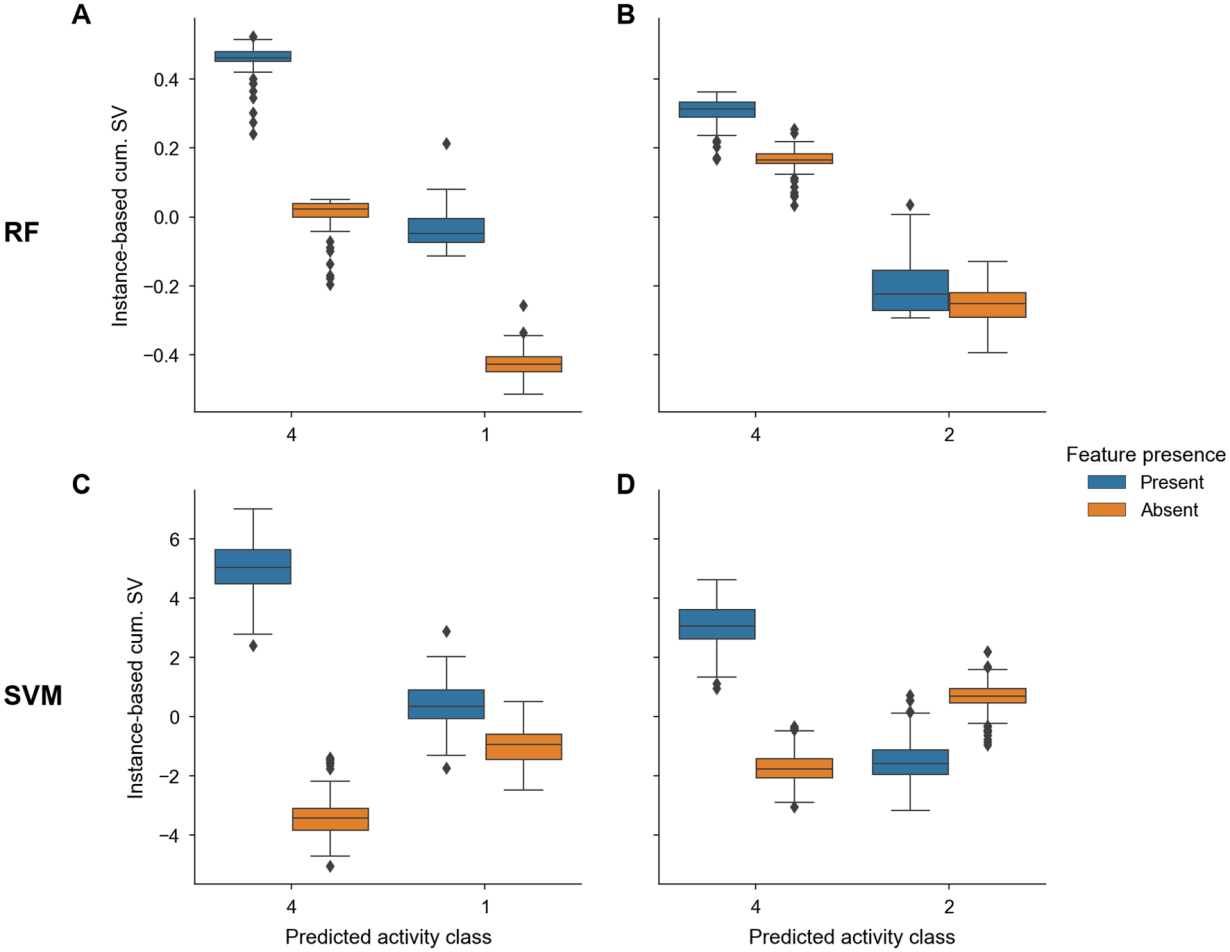
Instance-based cumulative Shapley values. In (**A,B**) and (**C,D**), boxplots show representative distributions of contributions of features that were present (blue) or absent (orange) in correctly predicted test compounds for RF and SVM, respectively. Results are shown for different pairs of activity classes (numbered according to Table 1) and models derived for the largest training sets. *SV* Shapley values.

Feature contributions to all correct predictions were quantified by calculating instance-based cumulative Shapley values (see “Methods”). For RF and SVM, different relative contributions of features that were present or absent in test compounds to the predictions were observed. Figure 2 shows two contribution distributions each for RF (Fig. 2A,B) and SVM (Fig. 2C,D) for four activity class pairs that were representative of predictions on all pairs. Shapley values for RF and SVM were of different magnitude. This was the case because Shapley values explained the probabilities of predictions for RF but accounted for the distance from the separating hyperplane for SVM.

For RF models, features present in test compounds from one activity class and absent in compounds from the other determined correct predictions (Fig. 2A), which was one of two prevalent contribution distributions. Alternatively, it was found that present and absent features in both activity classes comparably contributed to the correct prediction of their class labels (with positive and negative cumulative Shapley values, respectively) (Fig. 2B). For SVM models, present and absent features in one class supported and opposed the predictions, respectively, while present and absent features in the other class made only marginal contributions (with cumulative Shapley values close to 0) (Fig. 2C). These feature contribution distributions resulted in correct predictions because absolute cumulative Shapley values for present features were larger than for absent features. Alternatively, for other pairs, it was found that features present in the second activity class also supported correct predictions while features absent in this class were essentially neutral (or slightly opposed correct predictions) (Fig. 2D). Thus, for RF and SVM models, distinct relative feature contributions led to highly accurate predictions. Supplementary Fig. S2 shows that these characteristic feature contribution distributions evolved when training sets increased in size and the predictions reached high accuracy.

### Feature contribution patterns

The observed distributions were categorized as *feature* *contribution* *patterns* (FC_patterns). Accordingly, predictions were determined by features:

1. *Present* **or** *absent* in test compounds (observed only for RF),

2. *Present* **and** *absent* in test compounds (observed for RF and SVM),

3. **Only** *present* in test compounds (observed only for SVM).

To further analyze contribution patterns across all training set sizes and prediction trials, we defined a *feature* *contribution* *score* ${f}_{cs}$ as the difference between the median instance-based cumulative Shapley value for present features ${\widetilde{cs}}_{\mathrm{present}}$ and absent features ${\widetilde{cs}}_{\mathrm{absent}}$ contributing to the prediction of an activity class with a given model:

1 $${f}_{cs}={\widetilde{cs}}_{\mathrm{present}}-{\widetilde{cs}}_{\mathrm{absent}}$$

Accordingly, a positive feature contribution score indicated higher importance of present than absent features and a negative score the opposite. In addition, score values close to 0 indicated comparable contributions of features that were present or absent in test compounds.

**Figure 3:**
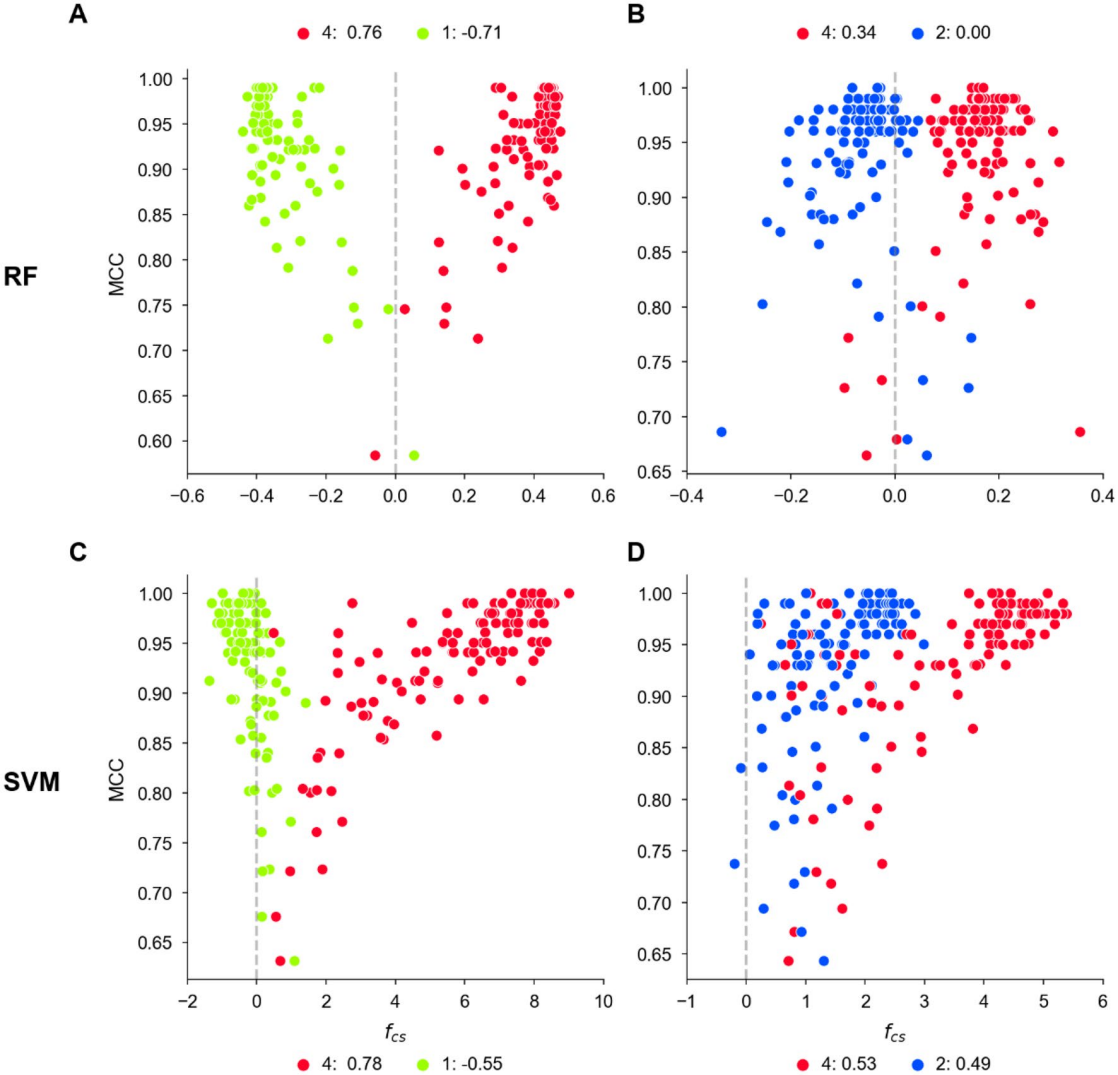
Feature contribution scores vs. prediction accuracy. In (**A–D**), scores ${f}_{cs}$ are plotted against the MCC values of the corresponding models for the same activity class pairs shown in Fig. 2. For each activity class (numbered according to Table 1), Pearson’s correlation coefficient (PCC) scores are reported in the legend.

For a given activity class pair and model, each pair of feature contribution scores was then associated with the MCC value of the prediction to determine the relationship between prediction accuracy and differentiation of features driving the predictions, as shown in Fig. 3 for the four activity class pairs from Fig. 2. The graphical representations revealed score distributions with characteristic shapes for the feature contributions in Fig. 2, which reflected different model phenotypes. Additional examples are provided in Supplementary Fig. S3.

For feature contribution scores and MCC values, Pearson’s correlation coefficient (PCC)^30^ was calculated for each activity class over all models, as reported in Fig. 3. A large absolute PCC value indicated that differentiation between contributions of present and absent features correlated with the predictive performance of the model, whereas a low PCC value indicated that differentiation between present and absent features was not relevant for the model’s performance. If *present* *or* *absent* features determined the predictions for an activity class pair (FC_pattern 1), the PCC value for one class was typically larger than $0.5$ and the other PCC value smaller than $-0.5$ for RF. If *present* *and* *absent* features were decisive (FC_pattern 2), the PCC value of one class was positive and the absolute PCC value of the other close to 0 for RF; for SVM, the PCC value for one class was typically larger than $0.5$ and the other PCC value smaller than $-0.5$. Finally, if *only* *present* features determined the predictions (FC_pattern 3), both PCC values were typically larger than $0.5$ (SVM).

### Correspondence between feature contributions

To further analyze feature contribution patterns, PCC values were also combined for each activity class pair (see Supplementary Methods):

2 $${c}_{RF, PCC}=\left|PC{C}_{1}\right|+\left|PC{C}_{2}\right|$$

3 $${c}_{SVM, PCC}=PC{C}_{1}+PC{C}_{2}$$

The two combined PCC patterns were defined to account for the different FC_patterns for RF and SVM described above in order to analyze RF and SVM models using the same threshold of 1 (details are provided in the Supplementary Methods).

**Figure 4:**
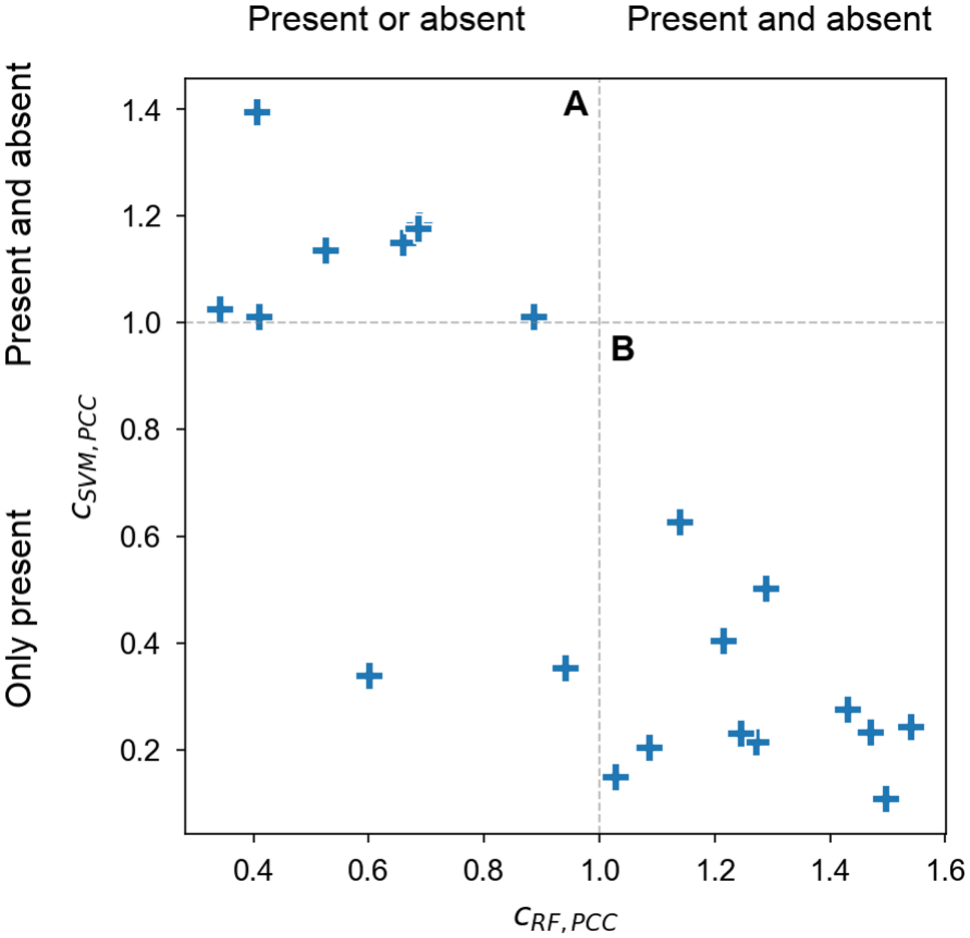
Comparison of combined Pearson’s correlation coefficients. Combined PCC values are compared for RF (x-axis) and SVM (y-axis). Each data point represents an activity class pair. Dashed lines indicate threshold boundaries (see Supplementary Methods). A and B mark areas of most frequently observed combinations of feature contribution patterns.

For both RF and SVM, combined PCC values larger than 1 were indicative of FC_pattern 2. Furthermore, for RF and SVM, values below 1 identified FC_pattern 1 and FC_pattern 3, respectively. In Fig. 4, combined PCC scores of all activity class pairs are compared for RF and SVM. For eight activity class pairs, SVM predictions were determined by FC_pattern 2 and RF predictions by FC_pattern 1 (area “A” in Fig. 4). In addition, for 11 other pairs, RF and SVM predictions were determined by FC_pattern 2 and 3, respectively (area “B”). The remaining two pairs combined FC_pattern 1 (RF) with 3 (SVM). For none of the activity class pairs, predictions were consistently determined by FC_pattern 2 shared by RF and SVM. Thus, the comparison of combined PCC values also revealed distinct feature contributions for RF and SVM and different learning characteristics.

### Relevance of individual features

Feature-based cumulative Shapley values (see “Methods”) were calculated to assess the importance of individual present or absent features for predictions. For each model and activity class, the top 15 present and absent features were identified and intersections for all activity class pairs determined. Feature overlap was only observed for features present in one and absent in the other class of a pair. Across all models, the feature overlap based on differently-sized training sets was larger for RF than SVM, with an intersection of 13 vs. six to seven top-ranked features, respectively.

**Figure 5:**
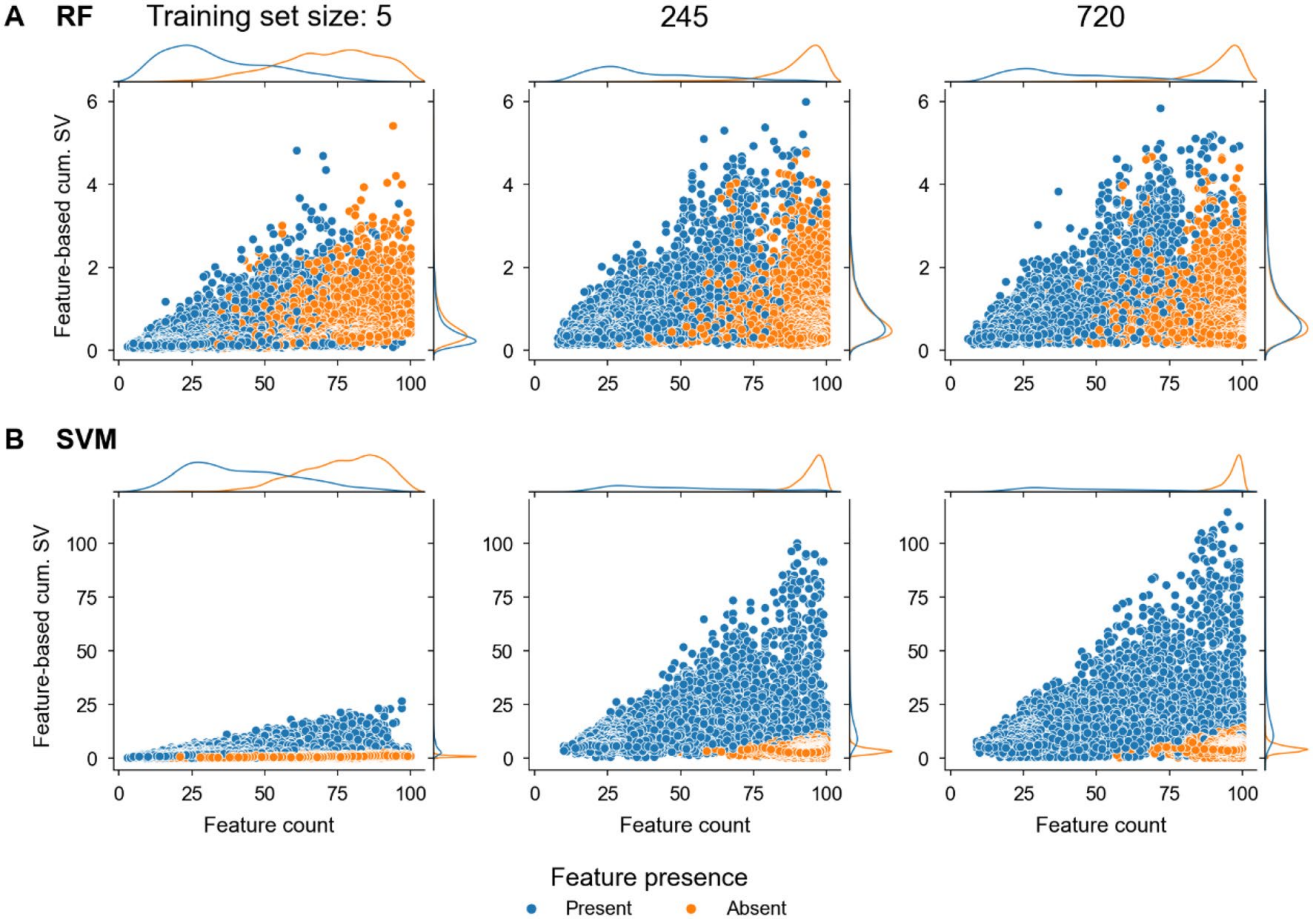
Contributions of individual features. Shown are distributions of cumulative Shapley values (SV) for the top 15 features of (**A**) RF and (**B**) SVM models derived from differently-sized training sets (y-axis) plotted against feature counts across all models. Blue and orange dots represent contributions of features that were present or absent in test compounds, respectively.

In Fig. 5, cumulative Shapley values for the top 15 features of all models derived from differently-sized training sets were compared to the occurrence of the feature in each model and the corresponding activity class. Only features making contributions to correct predictions were considered.

For RF models, cumulative feature contributions of present and absent features increased only slightly with increasing training set sizes. The importance of features generally increased with increasing feature counts, and features absent in test compounds made contributions comparable in magnitude to features that were present (Fig. 5A). In contrast, for SVM models, cumulative feature contributions substantially increased from small to medium-size training sets, but absent features made only minute or no contributions to correct predictions of models derived from training sets of increasing size (Fig. 5B), consistent with the analysis of FC_patterns discussed above. Taken together, these findings revealed distinct learning characteristics of RF and SVM.

### Feature mapping using Shapley values

**Figure 6:**
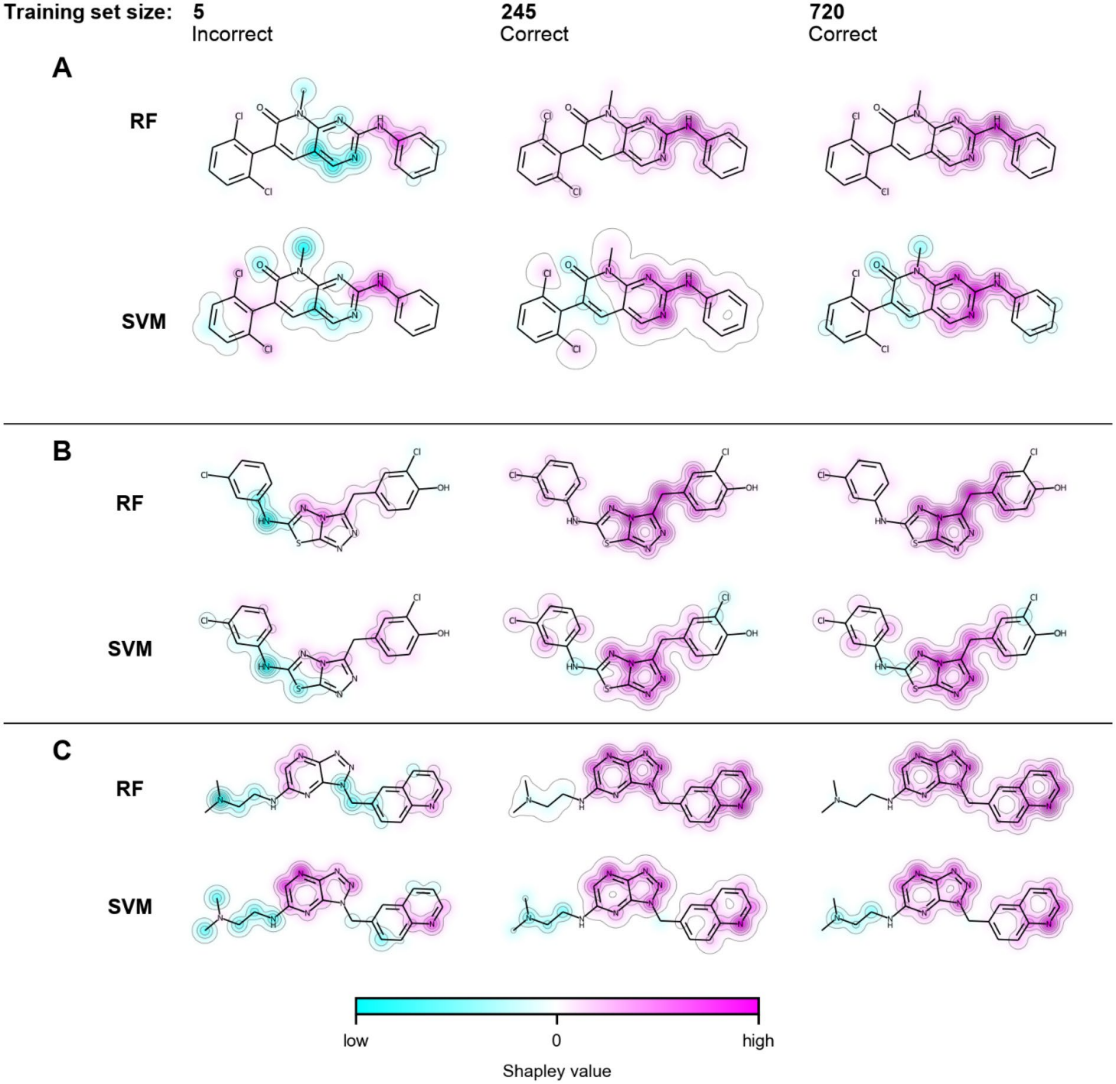
Feature mapping using Shapley values. In (**A–C**), Shapley values of features present in three exemplary test compounds with *Start: incorrect, End: correct* prediction pattern were assigned to the atoms forming these features and color-coded according to their cumulative atom-based contributions to predictions. The color spectrum from cyan over white to magenta indicates contributions opposing correct predictions (cyan, summed Shapley values < 0), neutral contributions (white, summed Shapley values ~ 0), and contributions supporting correct predictions (magenta, summed Shapley values > 0). For each test compound, feature mappings from three corresponding RF and SVM models based upon the smallest, and intermediate, and largest training set are compared.

Proceeding from models for the smallest to larger training sets, the prediction pattern *Start:* *incorrect,* *End:* *correct* was frequently observed (Table 2), as one might expect. For different models, instance-based Shapley values from features of test compounds that were first incorrectly and then correctly predicted were assigned to the atoms comprising each feature. As shown in Fig. 6 for representative compounds and models based upon the smallest, intermediate, and largest training sets, features determining correct predictions generally formed coherent substructures that largely overlapped for RF and SVM models (Supplementary Fig. S4 shows corresponding mappings for additional training set sizes). These observations were generally made for test compounds from all activity classes. Thus, despite their different feature contribution patterns, RF and SVM models ultimately prioritized features delineating corresponding substructures in correctly predicted compounds, hence providing a chemically intuitive explanation for consistently accurate predictions in the presence of distinct learning characteristics.

## Conclusion

In this work, RF and SVM models for activity-based compound classification were derived using differently-sized training sets that yielded very similar prediction patterns. These predictions were then analyzed in detail via an expanded Shapley value analysis scheme to explain and compare their origins. RF and SVM model decisions were largely determined by different contribution distributions of layered atom environment features present or absent in test compounds. These distributions resulted in different feature contribution patterns with varying correlations between feature contributions and prediction accuracy. RF models mostly relied on features that were present and/or absent in test compounds and consistently supported accurate predictions. By contrast, SVM models balanced contributions from features present in test compounds that supported correct predictions and absent features that mostly opposed correct predictions. Thus, despite comparably high prediction accuracy, RF and SVM displayed distinct learning characteristics. To complement the numerical analysis, Shapley value-based feature mapping on compound structures was carried out. Although RF and SVM feature contribution patterns mostly differed, features present in test compounds that determined accurate RF and SVM predictions delineated coherent and closely corresponding substructures, thus providing chemically intuitive explanations for these predictions. Taken together, the findings reported herein provide an in-depth view of learning characteristics of RF and SVM, which are among the most popular methods for molecular ML.

## Methods

### Activity classes

Compounds with activity against human targets were extracted from ChEMBL (version 30)^31^. Only compounds originating from assays with the highest confidence score of 9 and a molecular mass between 250 and 1000 Da were considered. Potential assay interference compounds^32^ identified with RDKit^33^ (version 2020.09.1) and aggregators^34^ were removed together with compounds violating a compendium of medicinal chemistry rules^35^. As potency measurements, numerically defined K_d_, K_i,_ or IC_50_ were required. Compounds were classified as active if they had (negative decadic logarithmic) K_d_, K_i_, or IC_50_ potency values above 5 (corresponding to 10 micromolar potency). Compounds with lower potency and compounds with inconsistent (active/inactive) potency annotations were discarded. On the basis of these rigorous activity data curation criteria, only 11 activity classes from ChEMBL met the pre-defined size threshold of 1000 compounds, seven of which were selected for our analysis, consisting of 1090–1988 compounds (Table 1), with on average 1341 compounds per class. The final selection of these seven classes was based on pairwise Tanimoto similarity calculations for each of the 11 classes (yielding intra-class similarity values) and combination of classes (inter-class values). On the basis of these calculations, four classes with largest differences between intra- and inter-class similarity values were omitted to limit structural heterogeneity potentially resulting in inhomogeneous prediction tasks (opposing meaningful comparison and explanation of classification models based on different algorithms).

### Machine learning models

#### Random forest

RF represents an ensemble of decision trees. Each tree is trained on a bootstrap sample of training compounds or the whole training set. At each node, only a subset of potential features is used to obtain the best separation of compounds with different class labels. RF models were built with *scikit-learn* (version 1.0.2)^36^. Hyperparameters including the number of trees (“n_estimators”: 100, 500), split quality criterion (“criterion”: gini, entropy), minimum number of samples per splits (“min_sample_split”: 2, 3, 4, 5), maximal number of features for achieving the best split (“max_features”: sqrt, log2), and potential bootstrapping (yes/no) of a sample of training compounds to derive each tree (“bootstrap”: True, False) were optimized using training data, as further described below.

#### Support vector machine

SVM is a supervised machine learning method that constructs a hyperplane in feature space maximizing the distance between different classes of objects. If the data cannot be linearly separated in the original feature space, a kernel function is applied to map the training instances to a higher-dimensional space where linear separation might become possible^37^. The kernel function calculates the similarity between two data points in the original feature space. Herein, the Tanimoto kernel^38^ was used, which is preferred for molecular similarity calculations. SVM models were built with *scikit-learn*. As hyperparameters, the cost “C” controlling the magnitude of permitted training errors (0.1, 1, 10, 50, 100, 200, 400, 500, 750, 1000, 2500, 5000, 750, 10,000) and the tolerance “tol” of the stopping criterion (10^–3^, 10^–2^, 0.1, 1, 2, 3) were optimized.

#### Molecular representation

Compounds were consistently represented using the extended connectivity fingerprint with bond diameter 4 (ECFP4)^39^ folded into 2048-bit vector generated with *RDKit*. ECFP4 captures layered atom environments in compounds and is a gold standard for molecular graph-based descriptors for molecular ML.

### Performance metrics

The predictive performance of the models on test sets was evaluated using three metrics generally applicable to test sets of any composition including Matthews Correlation Coefficient (MCC)^40^, balanced accuracy (BA)^41^, and the F1-score^42^, as defined below.

4 $$\mathrm{MCC}=\frac{\mathrm{TP}\times \mathrm{TN}-\mathrm{FP}\times \mathrm{FN}}{\sqrt{\left(\mathrm{TP}+\mathrm{FP}\right)\left(\mathrm{TP}+\mathrm{FN}\right)\left(\mathrm{TN}+\mathrm{FP}\right)\left(\mathrm{TN}+\mathrm{FN}\right)}}$$

5 $$\mathrm{BA}=\frac{1}{2}\cdot \left(\frac{\mathrm{TP}}{\mathrm{TP}+\mathrm{FN}}+\frac{\mathrm{TN}}{\mathrm{TN}+\mathrm{FP}}\right)$$

6 $$F1=\frac{2TP}{2TP+FP+FN}$$

TP, TN, FP, and FN abbreviate true positives, true negatives, false positives, and false negatives, respectively. BA and F1 values range from 0 to 1 and MCC values from 1 to −1. The selected metrics account for random prediction accuracy as a baseline for binary classification. Random accuracy is reflected by MCC and BA values of 0 and 0.5, respectively.

The different performance metrics were chosen to provide complementary information. BA is calculated as the average of sensitivity and specificity and thus represents the proportion of correct predictions. F1 constitutes the harmonic mean of precision and recall, thereby capturing recall performance and precision. However, F1 does not account for true negative (TN) predictions. Therefore, MCC is calculated that takes TP, TN, FP and FN predictions into account.

### Calculation protocol

RF and SVM models were derived to distinguish between compounds from different activity classes in a given pair. The models were trained and evaluated on identical data sets. Following generally accepted ML practice—and avoiding potential bias in model explanation by majority classes—all training sets were balanced. For each activity class pair and training sets size, ten independent trials with different randomly selected training, validation, and test sets were carried out. The size of the validation set was 20% of the corresponding training set size. Validation sets were used for hyperparameter optimization. Hence, the smallest of 12 training sets training set consisted of 10 (5 + 5) compounds with a corresponding validation set of 2 (1 + 1) compounds. Optimal hyperparameters were selected based on a random grid search with 50 trials. Therefore, models were ranked on the basis of MCC values. In each case, the model with hyperparameter settings yielding the largest MCC value for the validation set was selected as the final model. For small validation data sets, two hyperparameter sets might frequently yield the same MCC value. In this case, a loss function was calculated to serve as a “tie-breaker” to determine optimal hyperparameter settings. Herein, the log loss metric^43^ (*scikit-learn*) was used for RF, given in Eq. (7). In this function, $y\in \{0, 1\}$ represents the true label and $p$ the predicted probability $p=\mathrm{Pr}(y=1)$.

7 $${L}_{\mathrm{log}}\left(y,p\right)=-\left(y\cdot \mathrm{log}\left(p\right)+\left(1-y\right)\cdot \mathrm{log}\left(1-p\right)\right)$$

For SVM, hinge loss^44^ was applied, given in Eq. (8), with $d$ representing the output of the decision function, i. e., the distance to the hyperplane, and $y\in \{-1, 1\}$ referring to the true label.

8 $${\mathcalligra{l}}\,\,\left(d\right)=\mathrm{max}(0, 1-y\cdot d)$$

For each prediction trial, 100 compounds not used for training or validation were randomly selected from each activity class and combined to yield a constantly sized test set of 200 compounds per pair for model evaluation. Each test set was used to assess all models derived for training sets of increasing size, hence enabling the determination of prediction patterns.

### Shapley value analysis

The Shapley value concept originated from collaborative game theory^45^. Following this concept, the contribution of an individual player to the performance of a team is determined by distributing the result or “gain” of a game among all players according to their relative importance. In XAI, the *game* is understood as the *prediction* *task* *for* *a* *single* *instance* (here a compound) and each molecular representation *feature* corresponds to a *player*. The ability to quantify the contributions of features that are *present* or *absent* in an instance is of critical important aspect of the Shapley value formalism^3,4^, setting it apart from other feature weighting approaches. For large feature sets, the order-dependent systematic calculation of Shapley values becomes computationally prohibitive, requiring the introduction of local explanation models for most machine learning methods. However, for decision tree methods such as RF and for SVM using the Tanimoto kernel, exact Shapley values can be calculated using the TreeExplainer and SVETA methods to quantify feature contributions to the probability of a prediction and the distance to the hyperplane, respectively.

### Instance-based cumulative Shapley values

The cumulative Shapley value for features present or absent in an instance is calculated as the sum over all Shapley values for the instance. Instance-based cumulative Shapley values of all correctly predicted instances provide the overall importance of present and absent features for the predictions of a given model.

### Feature-based cumulative Shapley values

For correctly predicted instances, feature-based cumulative Shapley values are also calculated. For each instance, Shapley values are normalized such that the absolute sum of the values is equal to 1 (hence, Shapley values are divided by the absolute sum of all values). Then, for each predicted class, normalized Shapley values are summed for each individual feature that is present or absent. Normalization is performed to account for uncertainties and enable the comparison of cumulative contributions of individual features across different models.

## Supplementary Information

Supplementary Information.

## Data Availability

Compound classification calculations were carried out with public domain data and programs. Scripts for expanded Shapley value analysis are available from the corresponding author (bajorath@bit.uni-bonn.de) and will also be made available in an open access deposition with reference to this work.
