# Supplementary material for: Differences in learning characteristics between support vector machine and random forest models for compound classification revealed by Shapley value analysis
Source: Sci Rep. 2023 Apr 12;13:5983. doi: 10.1038/s41598-023-33215-x (PMC10097675; doi:10.1038/s41598-023-33215-x)
Supplement: Supplementary file 1 — Supplementary Information. [file 41598_2023_33215_MOESM1_ESM.docx]

# Supporting Information

# Differences in learning characteristics between support vector machine and random forest models for compound classification revealed by Shapley value analysis

Friederike Maite Siemers,^1^ Jürgen Bajorath^1*^

^1^Department of Life Science Informatics and Data Science, B-IT, LIMES Program Unit Chemical Biology and Medicinal Chemistry, Rheinische Friedrich-Wilhelms-Universität, Friedrich-Hirzebruch-Allee 5/6, D-53115 Bonn, Germany.

^*^Correspondence:

Tel. +49-228-7369-100, [bajorath@bit.uni-bonn.de](mailto:bajorath@bit.uni-bonn.de)

Supplementary Results

*Supplementary Figures S1-S4*

Supplementary Methods

*Combined Pearson’s correlation coefficients*

# Supplementary Results


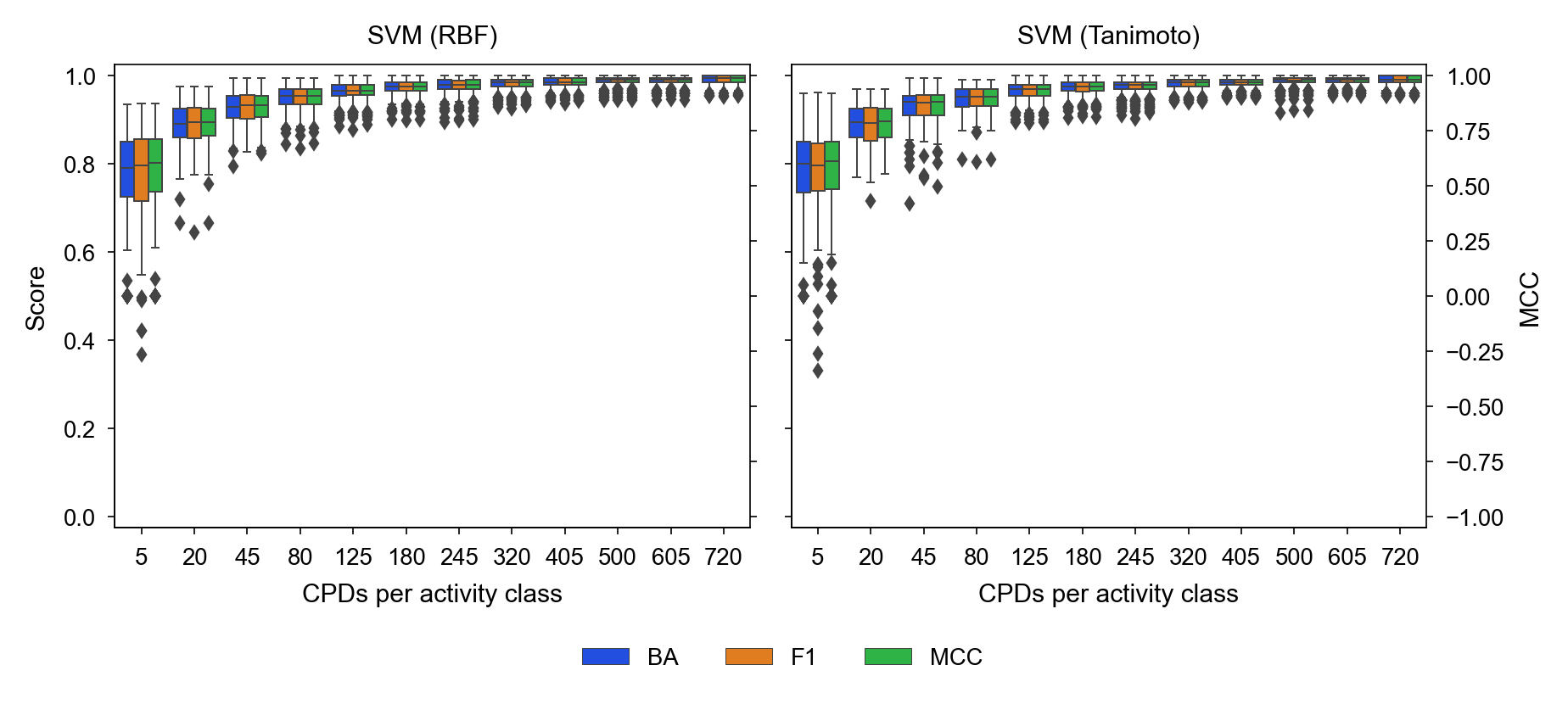


**Figure S1. Prediction accuracy.** SVM models built using the RBF (left) and Tanimoto kernel (right) on the basis of training sets of increasing size (CPDs per activity class; x-axis) are compared. The distribution of prediction accuracy values is reported in boxplots using BA and F1 scores (y-axis on the left) and MCC values (y-axis on the right). In boxplots, the median value is represented by the horizontal line, and the box defines upper and lower quantile. Upper and lower whiskers represent the maximum and minimum value, respectively. Diamond symbols mark statistical outliers.


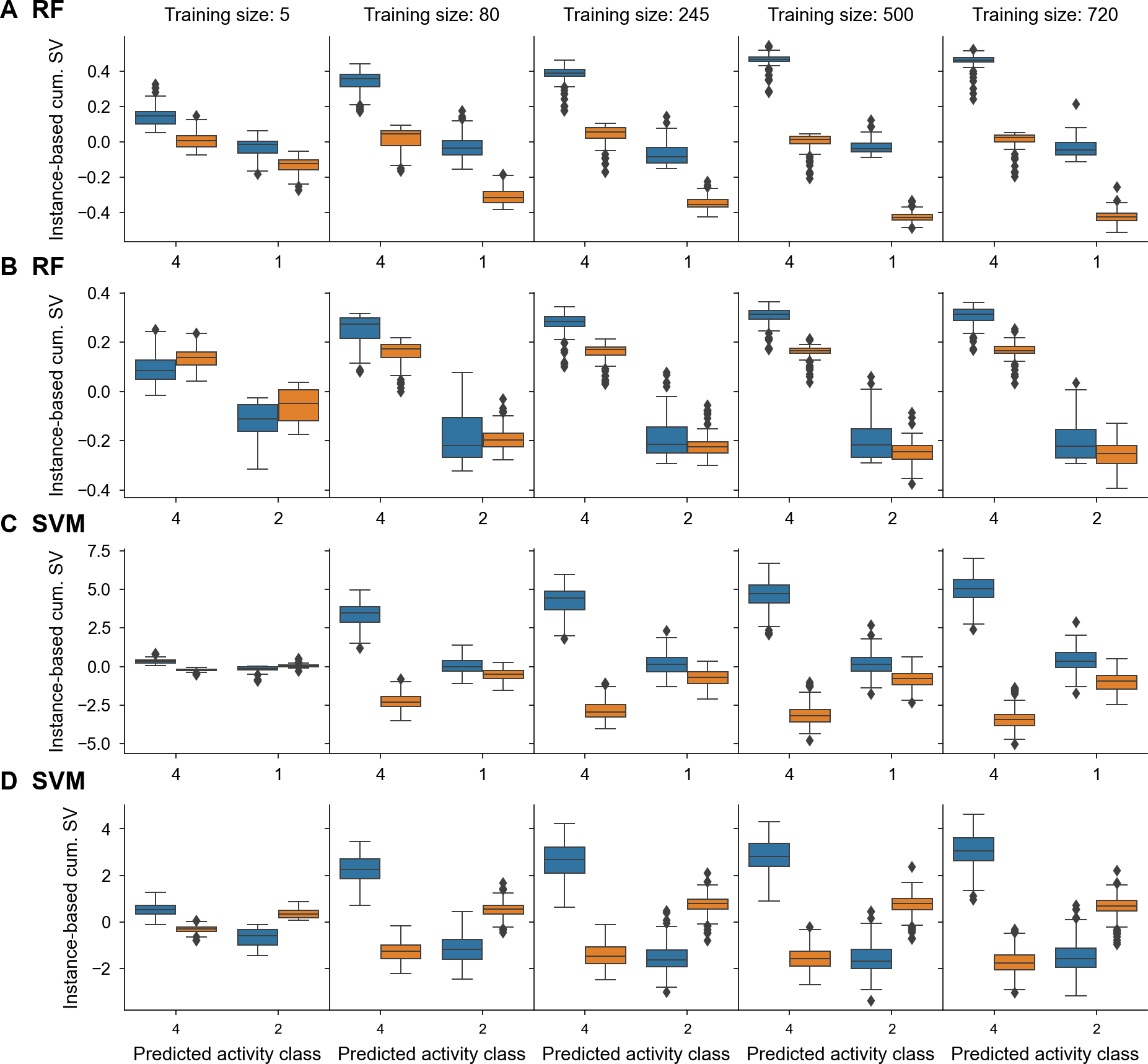


**Figure S2. Instance-based cumulative Shapley values for training of increasing size.**  In (**A**, **B**) and (**C**, **D**), boxplots according to Figure 2 show representative distributions of contributions of features that were present (blue) or absent (orange) in correctly predicted test compounds for RF and SVM, respectively. Results for the pairs of activity classes are shown for models derived from training sets of increasing size. SV denotes Shapley values.


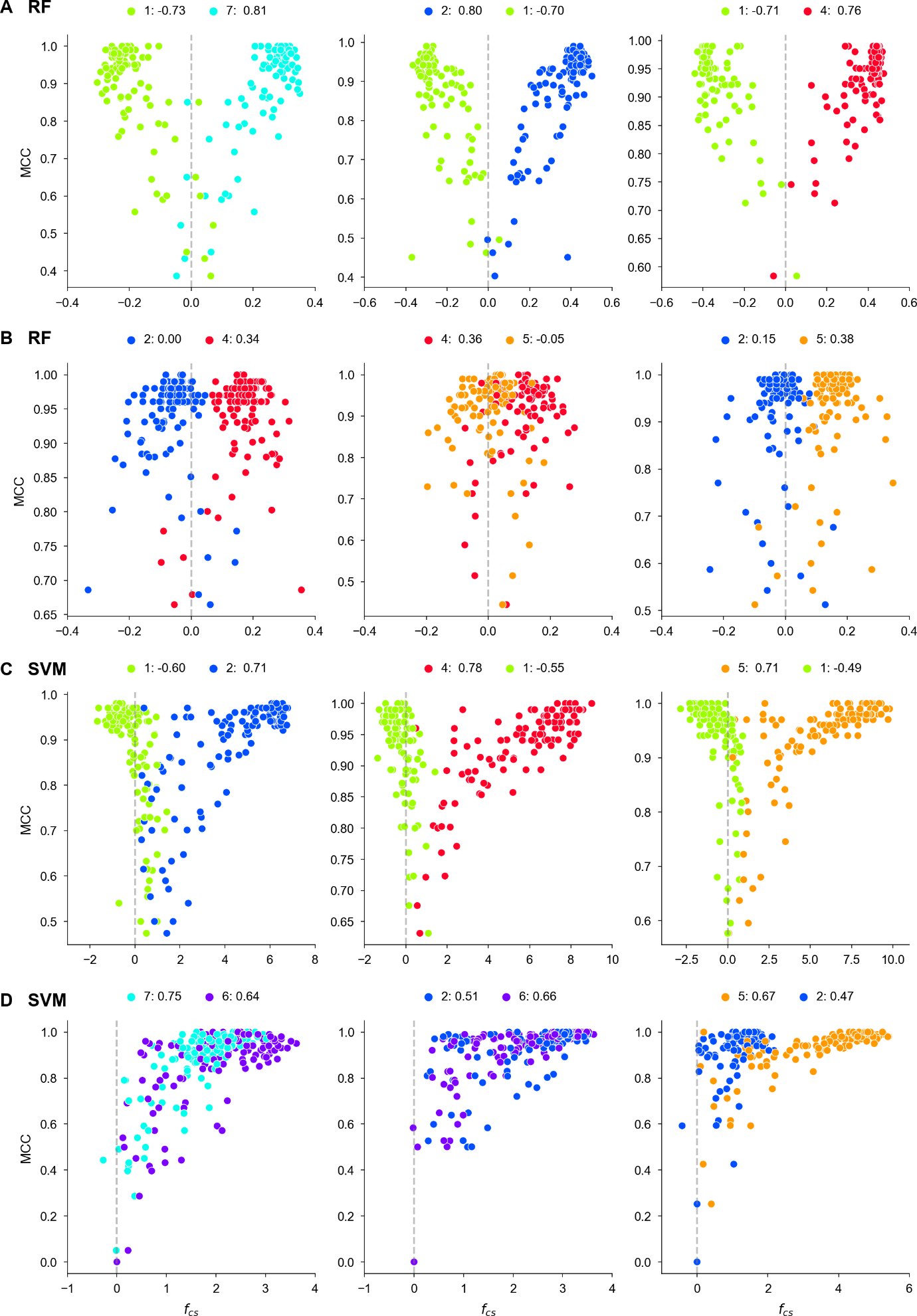


**Figure S3. Feature contribution scores vs. prediction accuracy.** Scores $f_{cs}$ are plotted against the MCC values of the corresponding. For each activity class (numbered according to Table 1), Pearson’s Correlation Coefficient (PCC) values are reported at the top. In (**A**)-(**D**), additional examples of target pairs are shown that resemble the score distributions in (A)-(D) of Figure 3, respectively.


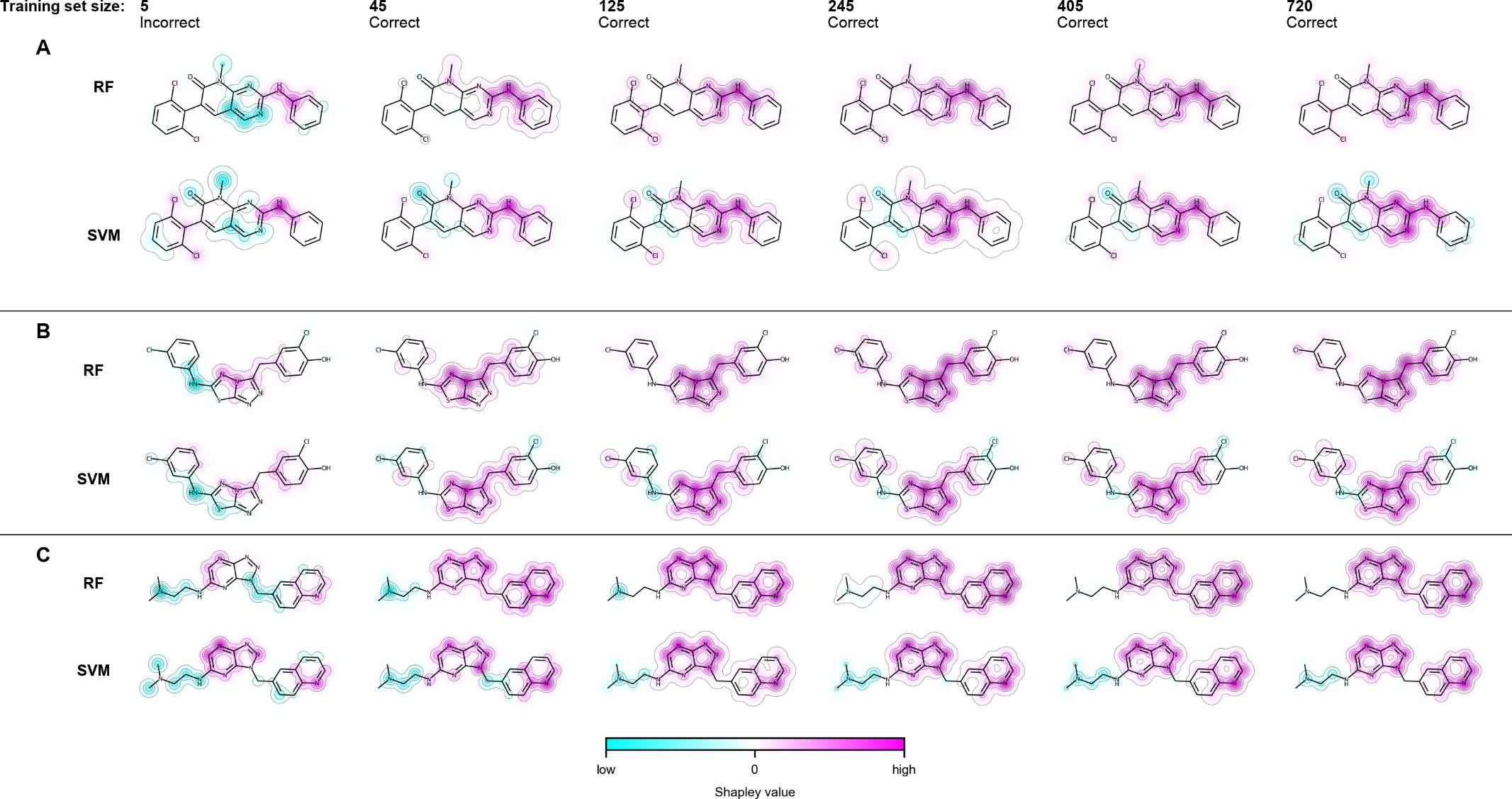


**Figure S4.** Feature mapping using Shapley values. In (**A**)-(**C**), Shapley values of features present in three exemplary test compounds with *Start: incorrect, End: correct* prediction patterns were assigned to the atoms forming these features and color-coded according to their cumulative atom-based contributions to predictions. The color spectrum ranging from cyan over white to magenta indicates contributions opposing correct predictions (cyan, summed Shapley values < 0), neutral contributions (white, summed Shapley values ~0), and contributions supporting correct predictions (magenta, summed Shapley values > 0). For each test compound, feature mappings from six corresponding RF and SVM models based upon training sets of increasing size are compared.

# Supplementary Methods

## Combined Pearson’s correlation coefficients

For the predictions, FC_patterns 1-3 were defined as reported in the text and can be represented based on the PCC of activity classes comprising a pair. The following thresholds are defined:

(A) FC_pattern 1(RF)

*Present or absent*

First activity class: $\mathrm{PCC}>0.5$

Second activity class: $\mathrm{PCC}<-0.5$

(B) FC_pattern 2 (RF)

*Present and absent*

First activity class: $\left| PCC \right|\approx0$

Second activity class: $PCC>0$

(C) FC_pattern 2 (SVM)

*Present and absent*

First activity class: $\mathrm{PCC}>0.5$

Second activity class: $\mathrm{PCC}<-0.5$

(D) FC_pattern 3

*Only present*

First activity class: $\mathrm{PCC}>0.5$

Second activity class: $\mathrm{PCC}>0.5$

Considering these boundaries, the *combined PCC scores* for RF $c_{RF,PCC}$ and SVC $c_{SVC,PCC}$ are defined as follows:

$$c_{\mathrm{RF},\mathrm{PCC}}=\left| \mathrm{PC}C_{1} \right|+\left| \mathrm{PC}C_{2} \right|$$

$$c_{\mathrm{RF},\mathrm{PCC}}=\mathrm{PC}C_{1}+PCC_{2}$$

These definitions yield the following upper and lower bounds resulting in threshold 1 for both combined PCC scores that differ between FC_patterns 1-3 for RF and SVC:

(E) FC_pattern 1 (RF)

Upper bound: $\left| {\mathrm{PCC}_{t}}_{1} \right|=\left| {\mathrm{PCC}_{t}}_{2} \right|=1\Rightarrow c_{RF,PCC}=2 (c_{RF,PCC}>1)$

Lower bound: $\left| {\mathrm{PCC}_{t}}_{1} \right|\overset{\to}{+}0.5\wedge\left| {\mathrm{PCC}_{t}}_{1} \right|\overset{\to}{+}0.5\Rightarrow c_{RF,PCC}\overset{\to}{+}1$ $(c_{RF,PCC}>1)$

(F) FC_pattern 2 (RF)

Upper bound: $\left| {\mathrm{PCC}_{t}}_{1} \right|\overset{\to}{0}0\wedge\left| {\mathrm{PCC}_{t}}_{2} \right|=1\Rightarrow c_{RF,PCC}\overset{\to}{-}1 (c_{RF,PCC}<1)$

Lower bound: ${\mathrm{PCC}_{t}}_{1}\overset{\to}{+}0.5\wedge{\mathrm{PCC}_{t}}_{2}\overset{\to}{+}0.5\Rightarrow c_{SVC,PCC}>1 (c_{RF,PCC}<1)$

(G) FC_pattern 2 (SVM)

Upper bound: $\mathrm{PC}C_{t_{1}}=1\wedge\mathrm{PC}C_{t_{2}}\overset{\to}{-}0\Rightarrow c_{SVC,PCC}<1 (c_{SVC,PCC}<1)$

Lower bound: $\mathrm{PC}C_{t_{1}}=-\mathrm{PC}C_{t_{2}}\Rightarrow c_{SVC,PCC}=0 (c_{SVC,PCC}<1)$

(H) FC_pattern 3 (SVM)

Upper bound: ${\mathrm{PCC}_{t}}_{1}={\mathrm{PCC}_{t}}_{2}=1 \Rightarrow c_{SVC,PCC}=2 (c_{SVC,PCC}>1)$

Lower bound: ${\mathrm{PCC}_{t}}_{1}\overset{\to}{+}0.5\wedge{\mathrm{PCC}_{t}}_{2}\overset{\to}{+}0.5\Rightarrow c_{SVC,PCC}>1 (c_{SVC,PCC}>1)$

The following annotation is used in the definition of upper and lower bounds:

$x\longrightarrow y$: value of $x$ converges to $y$

$x\overset{\to}{+}y$: value of $x$ converges to $y$ but $x>y$

$x\overset{\to}{-}y$: value of $x$ converges to $y$ but $x<y$
